# Carbon Catalyst Supports for Pt‐Based Polymer Electrolyte Membrane Fuel Cells: Porosity, Graphitization, and Chemical Modifications

**DOI:** 10.1002/advs.202508841

**Published:** 2025-10-27

**Authors:** Camille Roiron, Alessio Cosenza, Giovanni Ferro, Jiazhe Loki Chen, Hanson Wang, Plamen Atanassov

**Affiliations:** ^1^ Department of Chemical and Biomolecular Engineering National Fuel Cell Research Center University of California Irvine CA 92617 USA

**Keywords:** amorphous, carbon support, durability, graphitic, locality, PEMFC, porosity

## Abstract

Understanding the properties of carbon supports is essential for optimizing the performance and durability of Pt‐based catalysts in polymer electrolyte membrane fuel cells (PEMFCs). This review examines recent advances in the characterization and functional role of carbon supports, focusing on porosity, graphitization, and chemical modifications. Key methods used to assess the ordering of carbon phases and structural features are discussed, with attention to how these characteristics influence catalyst behavior. Particular emphasis is placed on the importance of fair comparison between Pt/C catalysts with different carbon supports, ensuring reliable performance assessments. The review then explores the interplay between graphitic and amorphous domains, highlighting how surface chemistry and chemical modifications can affect catalyst activity and stability. The impact of porosity and platinum nanoparticle locality is also examined, with special focus on how pore architecture can offer protection from ionomer‐induced poisoning and inhibit nanoparticle coalescence during operation. Throughout, the review identifies challenges in current characterization techniques and underscores the need for a holistic understanding of support architecture. Finally, it outlines design principles for next‐generation carbon supports that can better stabilize Pt nanoparticles and enhance the overall performance and durability of PEMFC cathodes.

## Graphical abstract

This review explores recent advancements in understanding the influence of carbon support properties on the cathode performance of proton exchange membrane fuel cells. It proposes a set of properties deemed critical for the development of next‐generation carbon supports for the oxygen reduction reaction in fuel cells.

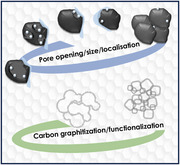

## 1 Introduction

Given the limited supply of natural resources and fossil fuels on our planet, alternative and renewable energy sources are preferred to meet society's needs.^[^ ^1^ ^]^ In particular, electrochemical energy conversion devices are attractive because they reduce emissions of greenhouse gases, such as CO_2_, which heavily contribute to global warming and climate change. Low temperature fuel cells, and in particular proton exchange membrane fuel cells (PEMFCs), are one such device that has been heavily researched over the past several decades.^[^ ^2^ ^]^ Much of this interest centers on catalysts for the cathodic oxygen reduction reaction (ORR), which is notorious for its sluggish kinetics causing overpotential losses during PEMFC operation.^[^ ^3^ ^]^ State‐of‐the‐art catalysts are based on Pt or Pt‐alloy nanoparticles supported on carbon materials.^[^ ^4^, ^5^ ^]^ In this work, all catalysts corresponding to this description will be designated as Pt/C materials. Various approaches to improving the intrinsic performance of Pt‐based nanoparticles have been explored; however, these studies are beyond the scope of this review, which primarily focuses on the carbon support.^[^ ^5^ ^]^

To serve effectively as a support in a Pt/C catalyst, a carbon material must exhibit high electrical conductivity and strong chemical stability under the harsh operating conditions of a PEMFC—namely, exposure to high acidity, a temperature of 80 °C, and a humid environment rich in water and oxygen. These requirements are generally met by thermally treated carbon materials, which can be classified based on their synthesis routes.^[^ ^6^ ^]^ Industrially produced variants include carbon black, furnace black, and acetylene black, while other classes encompass carbons obtained through pyrolysis of organic compounds, as well as graphene‐based materials and carbon nanotubes. At the nanoscale, these carbons exhibit crystallites of graphite (sp2 carbon) or graphite‐like crystallites that may be distorted due to the presence of defects and sp3 domains.^[^ ^7^, ^8^ ^]^ The extent of structural order sometimes informs the nomenclature; for instance, when these crystallites are well‐ordered over several nanometers and largely free of structural imperfections, the material is typically classified as graphitic carbon.^[^ ^9^ ^]^ In many cases, however, carbon materials display a combination of both ordered and disordered regions. The degree of crystallinity influences the type and density of surface groups.^[^ ^10^ ^]^ Long‐range, well‐ordered crystallites—without surface functional groups—have properties that differ significantly from those of the highly disordered amorphous regions.^[^ ^11^, ^12^ ^]^ In addition, strategies have been developed to modify the surface of the carbons by heteroatom doping.^[^ ^13^ ^]^ At a broader structural scale, carbon supports can exhibit a variety of morphologies. Porosity may arise either between the carbon primary particles or within them, resulting in a hierarchical network that includes macropores (>50 nm), mesopores (2–50 nm), and micropores (<2 nm).

**Figure 1:**
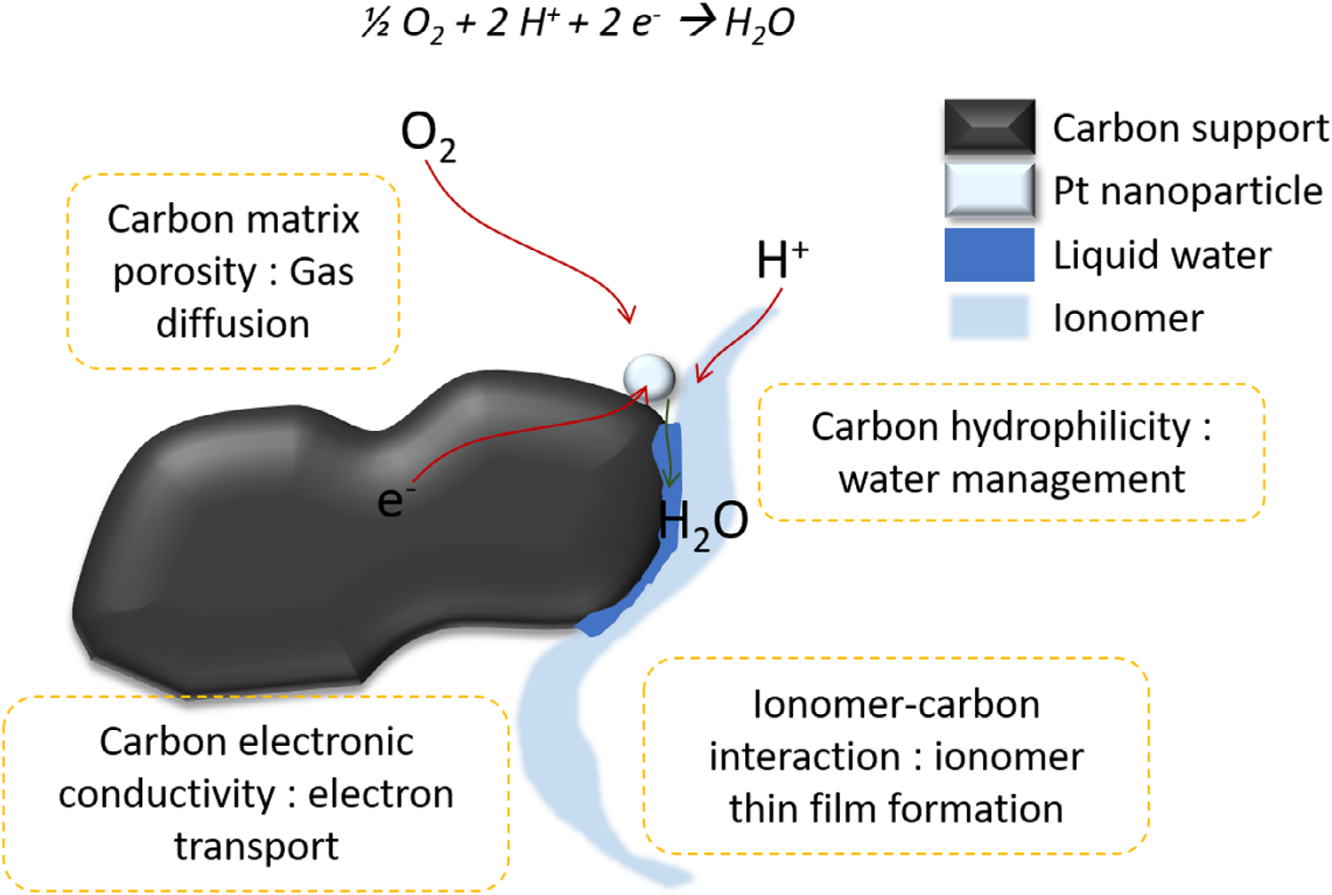
Schematic highlighting the role of the carbon support in the transport of ORR reactants and products.

At the cathode of a PEMFC, the ORR occurs at the surface of the Pt‐based nanoparticles; however, the other components of the catalyst layer are also crucial for achieving high performance and durability. As highlighted in **Figure** **1**, the carbon support plays a key role in each of the transport phenomena pertaining to the ORR. Protons are delivered to the active site via water and/or the proton‐conducting ionomer phase: a good dispersion of the ionomer on the carbon allows for the formation of a thin ionomer film on the carbon support surface.^[^ ^14^ ^]^ Oxygen is transported to the surface of the nanoparticles via the gas phase, encouraged by a porous network within the carbon support.^[^ ^15^ ^]^ The electrons flow toward the active site via the carbon support, which must have sufficient conductivity to not be the limiting factor. Finally, the relative hydrophobicity of the carbon is essential to prevent oxygen diffusion limitations caused by the accumulation of water produced during the ORR.^[^ ^15^ ^]^

The durability of the cathode catalyst layer is a major stake for the development of state‐of‐the‐art PEMFCs^2^. Contained within that is the longevity of the carbon support, which remains an important yet challenging task. A special accelerated stress test (AST)—consisting of triangular waves between 1.0 and 1.5 V of cell potential—has been developed to target the carbon support by inducing carbon corrosion.^[^ ^16^, ^17^ ^]^ Under normal fuel cell operation, this condition would only occur during uncontrolled startup or shutdown events.^[^ ^18^ ^]^ System solutions have been devised to prevent these events by controlling the gas flow and avoiding operation of the fuel cell in non‐steady‐state gas composition. Still, the resistance to corrosion of the carbon supports remains a relevant metric. Intense carbon corrosion, such as that generated by the carbon AST, oxidizes the support completely to gaseous CO_2,_ triggering local collapse of the carbon structure and crumbling of the catalyst layer.^[^ ^19^ ^]^ On the other hand, milder corrosion, occurring at lower potentials due to the presence of Pt nanoparticles, can modify the surface chemistry of the carbon, altering hydrophobicity and interaction with the ionomer.^[^ ^20^, ^21^, ^22^ ^]^ In contrast to the carbon, degradation of the Pt‐based particles is better studied with a milder AST protocol (0.6–0.95 V of cell potential). Mechanisms for nanoparticle aging include agglomeration, detachment and Ostwald ripening (dissolution of Pt and redeposition onto bigger particles), all of which contribute to losses in electrochemically active surface area (ECSA).^[^ ^23^, ^24^ ^]^ Particle agglomeration during aging can be influenced by the chemistry of the carbon support, depending on whether it can provide anchoring or favor the lateral movement of the nanoparticles. The presence of ionomer in the vicinity of the nanoparticles is expected to have two effects: a decrease in the local pH^[^ ^25^, ^26^ ^]^ and poisoning of the Pt by the pendant sulfonic groups.^[^ ^27^, ^28^ ^]^ The concern around increased acidity is heightened for Pt‐alloy systems containing transition metals due to their tendency to dealloy.^[^ ^29^ ^]^ The morphology of the carbon can provide some shielding to the nanoparticles, reducing direct contact with the ionomer, but this may come at the expense of proton accessibility to the active sites.^[^ ^30^, ^31^ ^]^

The multiple roles of the carbon support in the catalyst layer lead to interconnected effects, making it complicated to identify the properties of the ideal ORR carbon support. The present review does not claim to have found said ideal carbon support but aims at compiling and sorting the knowledge of the community regarding the properties of the carbon support. We avoid listing all the different novel carbons in a process‐based approach and instead focus on the desirable properties of a carbon support, while highlighting the fundamental knowledge gap and recommending methods to address it.

## 2 Methods for the Study of Carbon Support Properties

### 2.1 Ordering of the Carbon Phase(s)

**Figure 2:**
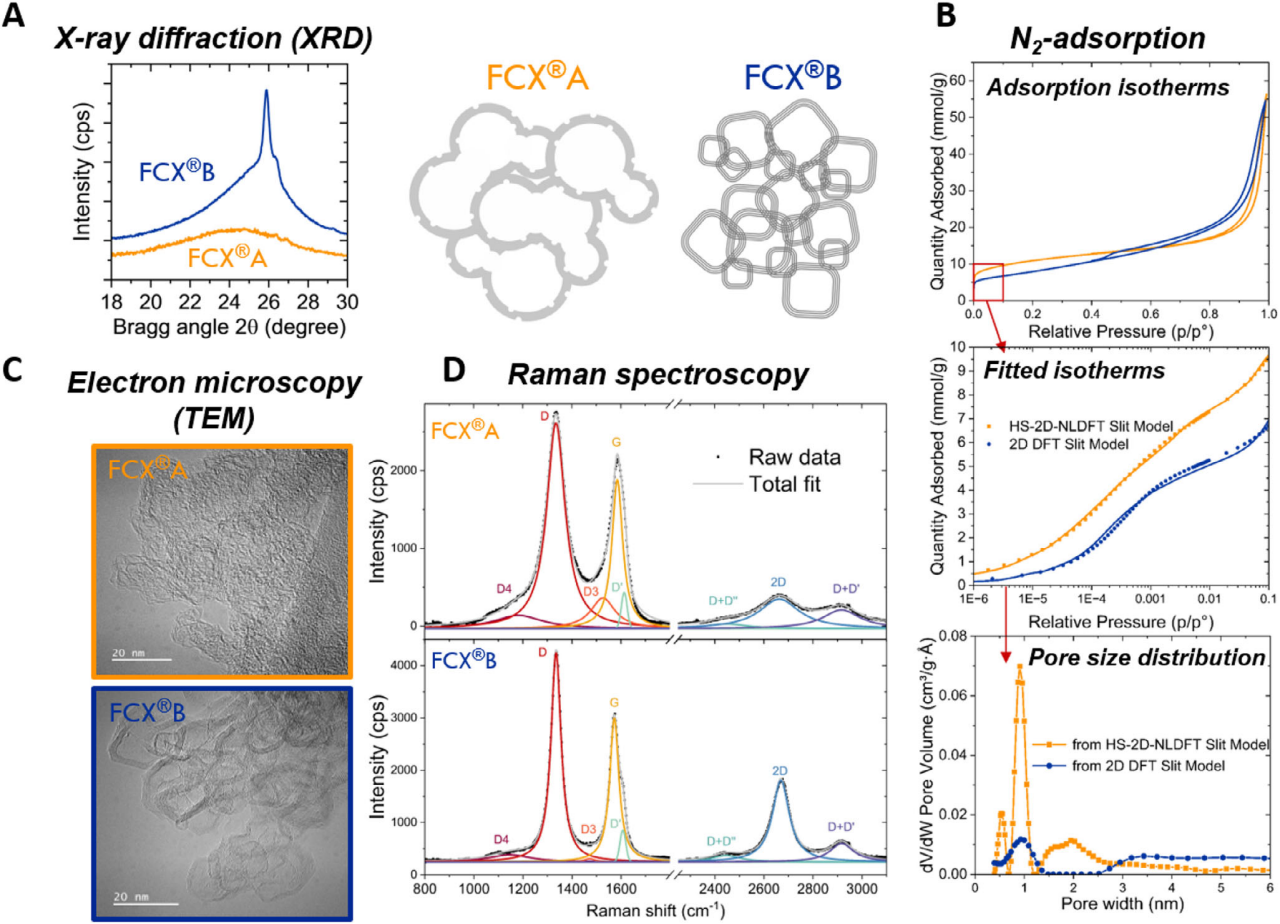
Nonexhaustive collection of techniques used to characterize the carbon support structure. A) X‐ray diffraction (XRD), B) Nitrogen adsorption isotherms and pore size distribution modelling using NLDFT models: the slit models HS‐2D‐NLDFT (mod255.df2) and 2D DFT (mod000.df2) for FCXA and FCXB, respectively. C) Transmission electron microscopy (TEM) and D) Raman spectroscopy. By combining various techniques a schematic representation of the carbon material can be built. Adapted with permission. [22] © 2024, Wiley‐VCH GmbH.

Carbon materials can exhibit a wide spectrum of structural organization, ranging from highly ordered graphitic forms to entirely amorphous structures, as well as various intermediate situations. A common approach to assess the degree of crystallinity is through X‐ray diffraction (XRD), where the (002) reflection, typically observed near 2θ ≈ 26°, serves as a key indicator of structural ordering. An example of X‐ray diffraction patterns for different carbon structures is given in **Figure** **2**A.

In well‐ordered graphite, this peak appears sharp and is centered ≈26°, corresponding to an interlayer spacing (d_002_) of ≈0.335 nm. In such cases, the Scherrer equation can be reliably applied to estimate the out‐of‐plane crystallite size, provided the assumptions regarding shape and strain are satisfied. However, in disordered or amorphous carbons, the (002) peak broadens significantly and shifts toward lower angles. This shift is attributed to an increase in the average interlayer distance, which may result from local stacking faults, microstrain, or an overall lack of coherent organisation. These structural irregularities produce a wide distribution of d‐spacing values that render the Scherrer equation inapplicable, as it assumes a well‐defined crystalline domain.^[^ ^32^ ^]^ Notably, discrepancies can be observed between the stacking number measured by microscopy and the crystallite size calculated by the Scherrer equation.^[^ ^33^, ^34^ ^]^

In many practical carbon materials, a coexistence of ordered and disordered domains is observed. Transmission electron microscopy (TEM) often reveals nanocrystalline regions with graphite‐like characteristics chaotically interconnected through disordered domains or embedded within amorphous regions.^[^ ^35^ ^]^ The term “graphite‐like” refers to short‐range stacked graphene layers that retain some crystallographic order while being perturbed by various types of defects.^[^ ^11^ ^]^ These include substitutional heteroatoms, edge functional groups, vacancy defects within the basal planes, interspersed sp^3^‐hybridized regions and bent regions. Such defects introduce microstrain and disrupt the planarity of graphene layers, leading to local structural distortions within individual crystallites. Because the basal planes inside these crystallites are imperfectly stacked or slightly shifted, a condition known as turbostratic disorder, the crystallites themselves fail to align coherently within the primary carbon particle.

The shape and position of the (002) peak, therefore, capture both crystallite size and microstrain information. In partially ordered materials, peak broadening may result from either or both factors. Microstrain, in particular, arises from intra‐layer distortions, leading to a range of inter‐layer distances within individual crystallites. When both amorphous and graphitic components are present, the diffraction profile may exhibit a convolution of features—namely, a broad component occurring at 2θ < 26° and a sharper contribution closer to the ideal graphite position.^[^ ^36^ ^]^ In such cases, deconvolution of the overlapping signals is recommended to separate the structural contributions and obtain a more accurate understanding of the material's microstructure.

Raman spectroscopy is another widely used tool for characterizing the organization of the carbon structure. It is important to highlight that Raman spectroscopy captures contributions from each symmetric vibration of the chemical bonds and should therefore not be oversimplified as a ratio of ordered to disordered carbon.^[^ ^11^ ^]^ Figure 2D offers an overview of the different Raman features obtained for two different carbons. A detailed analysis of the spectra can help identify the type of crystallization and track relative changes across a series of materials.^[^ ^37^, ^38^ ^]^ In carbon structures containing nanocrystalline graphite, the intensity ratio of the D and G bands is commonly used to estimate the in‐plane crystallite size (La). The appearance of a sharp 2D band indicates a higher degree of long‐range order and provides complementary structural information beyond that offered by the D/G ratio.

Transmission electron microscopy (TEM) is widely used to characterize the morphology of different carbon phases. At sufficiently high resolution, graphitic zones become visible, and the relative positions of amorphous and graphitic regions can be observed, offering insight into the morphology of the support (Figure 2C).

### 2.2 Structural Properties

The morphology of carbon materials can, to some extent, be assessed by imaging. TEM images are a 2D projection of the material that provides good contrast for the ordered features but does not offer a 3D view of the material's morphology. A more comprehensive characterization can be achieved with TEM tomography. This technique provides information on pore size, shape, and opening, as well as the locality of Pt‐based nanoparticles relative to the pores.^[^ ^39^ ^]^ Scanning transmission electron microscopy with a secondary electron detector (STEM‐SE) can provide an alternative to TEM‐tomography when the 2D projection obtained by TEM (displaying all nanoparticles) is compared to the surface‐sensitive secondary electron image (displaying only the particles on the outer surface).^[^ ^22^, ^40^ ^]^ This technique does provide the same resolution as TEM tomography but allows a qualitative assessment of the locality of the Pt nanoparticles.

For a general assessment of the porosity of the carbon support, the reference method is nitrogen adsorption isotherms. The main parameter extracted from the isotherm is the specific surface area calculated by the Brunauer–Emmett–Teller method (BET). For a more detailed description of the pore structure, the Barrett–Joyner–Halenda method (BJH) gives a pore size distribution in the range of 4–50 nm. When complemented by a nonlocal density functional theory (NLDFT) model, additional information is obtained on pores ranging from 0.5 to 20 nm. This method involves fitting the adsorption isotherm and selecting an appropriate pore model that matches the isotherm shape; only in this way can a meaningful pore size distribution be derived and insight gained into the material's pore architecture. For example, in Figure 2B, both isotherms are fitted with a slit model, indicating small pores opening in the shape of slits. Similar caution must be exercised when using the t‐plot method to calculate the total microporous volume or surface. While many resources analyze the nitrogen adsorption isotherms of bare carbon supports, their interpretation becomes more complicated when applied to carbons containing Pt nanoparticles.^[^ ^41^ ^]^ All the metrics related to N_2_‐adsorption studies are normalized by the mass of material. In Pt/C materials, a significant fraction of the mass is occupied by the nanoparticles, and this method does not allow for the separation of Pt and carbon surface contributions.

While N_2_ adsorption isotherms are often used primarily for surface area and pore structure analysis, using other adsorbates like water can help assess other relevant aspects, such as surface hydrophilicity and moisture interaction, both before and after nanoparticle introduction.^[^ ^40^, ^41^ ^]^

While adsorption techniques probe the porosity, it is possible to probe the size and structure of the aggregates of carbon primary particles using small‐angle X‐ray scattering (SAXS).^[^ ^42^ ^]^

### 2.3 Fair Comparison of Pt/C Catalysts with Different Supports

**Figure 3:**
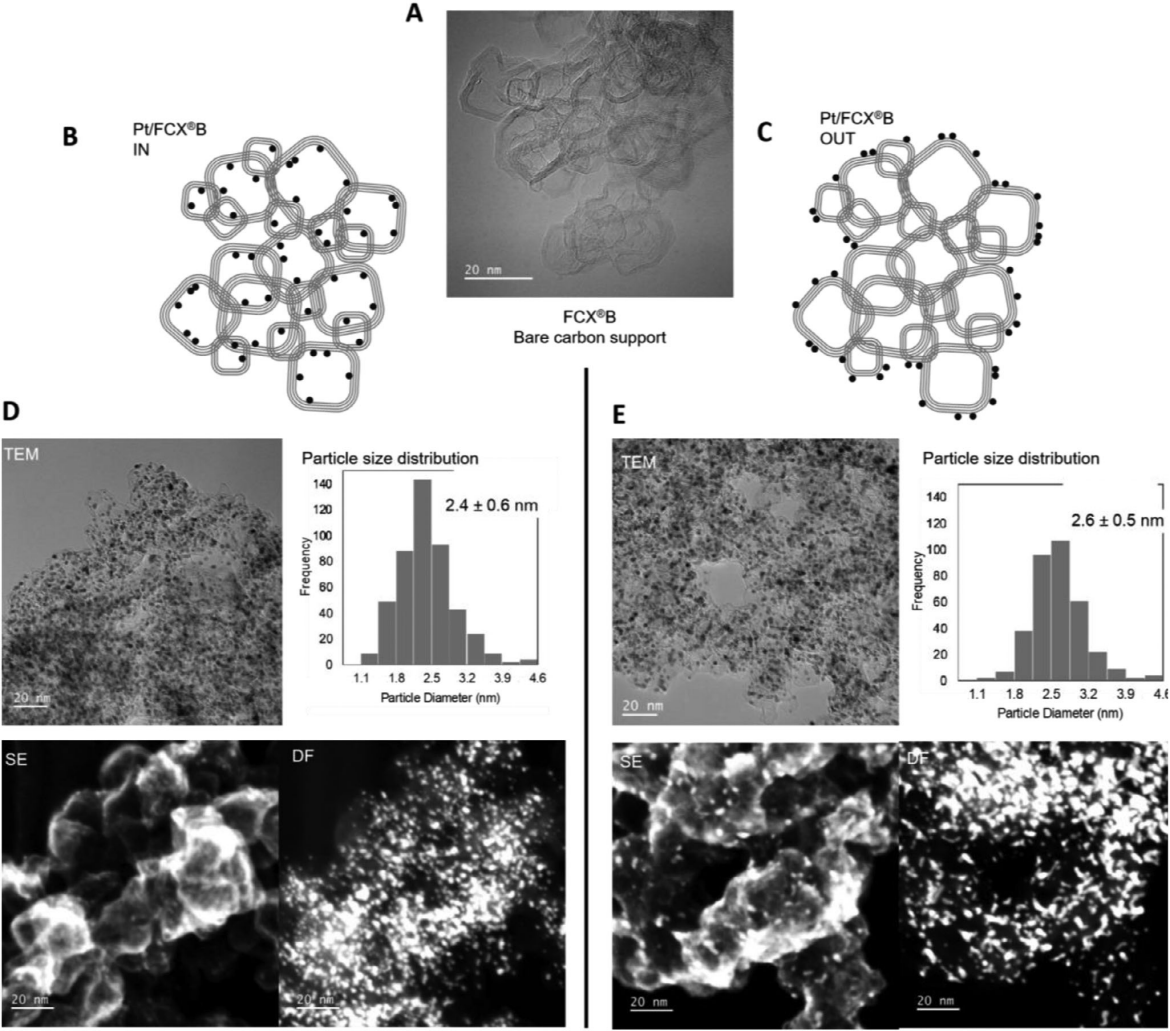
A) TEM image of the bare FCXB carbon support. B,C) Schematic representation of the locality of the nanoparticles deposited predominantly inside (B) and outside (C) of the mesopores of the carbon support and D,E) characterization of the materials by TEM (left‐top), particle size distribution (right‐top), Surface‐sensitive secondary electron (SE) microscopy capturing only the Pt nanoparticles located on the exterior surface of the carbon supports (left‐bottom) and corresponding dark‐field (DF) image confirming the presence of all Pt nanoparticles in the field of view (right‐bottom). Adapted with permission. [22]. © 2024, Wiley‐VCH GmbH.

When evaluating the influence of specific carbon properties on catalyst activity and durability, a common challenge is ensuring that only one variable is being assessed at a time. If materials differ too broadly, meaningful comparisons become difficult. For instance, comparing carbons produced using different synthesis methods or sourced from different manufacturers often results in simultaneous variations in graphitic content, porosity, and surface chemistry. This makes it difficult to isolate the impact of any single property on performance. In addition, to compare catalysts, the incorporation of Pt nanoparticles is needed. Some studies use and compare commercial catalysts with Pt already deposited. In this situation, great care must be taken that the Pt nanoparticles are similar enough. Indeed, a smaller particle size at the beginning of life strongly promotes particle growth and activity loss, independently of the carbon support.^[^ ^43^ ^]^ The specific activity of the Pt nanoparticles also decreases with the initial size.^[^ ^44^ ^]^ As evidenced in **Figure** **3**, preparing the Pt particles in‐house can help ensure comparability between them. However, the chemistry of the carbon surface can affect the nucleation of the particles and generate different sizes and dispersion of the particles on the carbons, even with an identical in‐situ synthesis procedure. It is possible that synthesis parameters would have to be adapted to generate comparable materials.^[^ ^22^ ^]^ One alternative to this is the preparation of Pt nanoparticles ex‐situ followed by their subsequent deposition on different carbon supports. Even then, care must be taken that the targeted loading is achieved since the anchoring of the particles on the support can vary for different supports.^[^ ^45^ ^]^ Furthermore, this ex‐situ method can make it difficult to incorporate particles into the pores of the supports. To achieve a specific locality of the nanoparticles on the carbon, different synthesis methods can be employed as long as particle size and crystallinity can also be controlled. Figure 3 illustrates such a material set with similar particle size prepared by two different synthesis methods to achieve different localities. When locality, Pt loading, particle size, and crystallinity are not carefully controlled, materials can differ in multiple aspects, making it necessary to perform cross‐study comparisons to deconvolute the individual effects of each property.

## 3 Role of the Chemistry and Graphitization

### 3.1 Graphitic and Amorphous Domains

The carbon supports can have many different arrangements of graphitic crystallites and amorphous zones. Differences are expected in their ability to: anchor platinum and resist corrosion to prevent particle agglomeration and detachment, respectively.

At the edges of the crystal planes, the carbon atoms are sp3‐hybridized and are less chemically stable due to the coordination loss and the presence of functional groups. The defective edges of the crystallites are preferentially corroded, as demonstrated in a work by Cherstiouk et al. studying carbon materials from graphitized and amorphous families.^[^ ^12^ ^]^ Similarly, it was measured that carbon corrosion aging generates a loss of amorphous carbon preferentially compared to the graphitic zones.^[^ ^37^ ^]^ This gives the illusion of a more graphitic carbon after AST due to the removal of amorphous carbon toward CO_2_.

Graphitic carbons have shown higher stability in thermal and electrochemical corrosion.^[^ ^12^, ^38^ ^]^ New carbon supports with high graphitic contents have been developed with increased durability.^[^ ^46^, ^47^, ^48^, ^49^ ^]^ However, surface modifications such as nitrogen incorporation or organic modifiers are needed to anchor the nanoparticles at the surface.^[^ ^49^, ^50^ ^]^ These materials systematically display better durability and retention of ECSA upon aging.^[^ ^38^, ^46^, ^47^, ^48^ ^]^ For that reason, it is accepted by the community that graphitic carbons are preferred for the durability of catalysts. However, in most cases, the improved durability cannot be solely attributed to the decreased detachment mechanism due to less corrosion. For example, Wang et al. demonstrate the benefits of a highly graphitic support even when cycling between 0.6 and 1.0 V.^[^ ^48^ ^]^ In that regime of degradation, Ostwald ripening is expected to be the main mechanism and, therefore, the low ECSA loss observed implies the mitigation of all the degradation mechanisms, including Ostwald ripening. This would indicate that the use of a graphitic support would prevent the dissolution of the platinum, and this phenomenon requires more targeted investigations.

Additionally, different durability has been observed among predominantly amorphous carbon supports. For example, in a 1.0–1.5 V AST, Ketjenblack EC300J showcased less degradation than Vulcan XC72 despite both having a low degree of graphitic order.^[^ ^37^, ^51^, ^52^ ^]^ The study by Zana et al. studies the corrosion by Raman spectroscopy with identical particles and deposition processes, ensuring a direct comparison of the carbon structures.^[^ ^37^ ^]^ After the AST, with or without Pt, C═O groups are formed on the Vulcan but not on the Ketjenblack, despite having a very similar Raman signature before aging.

The role of Pt nanoparticles to catalyze carbon corrosion seems to vary from one support to another. In separate studies by Cherstiouk et al. and Zana et al., several carbon supports are corroded with and without Pt with identical Pt nanoparticles.^[^ ^12^, ^37^ ^]^ The presence of Pt nanoparticles has been found to be beneficial for some carbon supports, while accelerating degradation in others. This effect does not appear to correlate directly with the degree of graphitization or the specific type of carbon used. Further studies are needed to fully understand the nature of this interaction.

Structural parameters describing the degree of graphitization—such as L_a_, L_c_, and d‐spacing—are often reported and calculated through standard methods like Raman spectroscopy and XRD. However, the complexity and heterogeneity of carbon's crystalline structure make these values simplifications at best. They represent average or “apparent” parameters that do not capture the complexity and uniqueness of the actual materials. Drawing reliable conclusions about the crystalline architecture requires a combined approach: high‐resolution imaging must be supported by spectroscopic and diffraction data, with careful attention paid to both structural disorder and the degree of amorphicity. A more nuanced assessment of these features is essential for understanding their influence on catalyst performance and durability.

These structural descriptors contain information on corrosion mechanisms and nanoparticles anchoring to the support. For example, the pits formed by the edges of adjacent graphite crystallites can promote anchoring of nanoparticles, but these edges are more reactive and thus more vulnerable to corrosion.^[^ ^53^ ^]^ Carbon materials with large in‐plane crystallite sizes (La) are characterized by extended graphitic domains with a low density of edge and defect sites.^[^ ^54^, ^55^ ^]^ While such ordering enhances resistance to electrochemical corrosion, it simultaneously reduces the number of anchoring sites available for nanoparticle deposition. As a result, the supported nanoparticles tend to be less uniformly distributed, which negatively impacts catalyst utilization and long‐term stability. Conversely, carbons with smaller La and Lc values expose a higher concentration of edge planes and structural defects that promote a more uniform Pt nanoparticle coverage.^[^ ^56^ ^]^ However, these reactive sites are also more susceptible to oxidation under operating conditions, accelerating carbon corrosion and leading to faster catalyst degradation.^[^ ^39^ ^]^ This trade‐off between stability of the carbon framework and the effectiveness of nanoparticle dispersion is a limitation in the design of carbon‐supported catalysts.

The crystalline structure cannot be studied completely independently from the porous structure of the carbon support. Indeed, micropores often originate from the narrow spaces between small, tightly packed crystallites, where a high density of edge sites—often carrying oxygen‐containing groups—can make these regions more prone to corrosion. In the case of graphitic mesopores, different configurations can be imagined with the walls of the pores consisting of either basal planes or edges. In some cases, micropores may serve as entry points to these inner mesoporous networks.^[^ ^57^ ^]^ To enhance the stability of such pore entrances, which tend to be more reactive, introducing heteroatoms other than oxygen through chemical doping could be a promising direction. These structural distinctions highlight the need for refined structural characterization tools and metrics. Looking ahead, a more detailed classification of carbon crystallinity—beyond simple averages—will be key to designing supports with tailored anchoring behavior and controlled corrosion profiles. Developing such insight will be critical for advancing the next generation of durable, high‐performance fuel cell catalysts.

### 3.2 Chemical Surface Modifications

The surface chemistry of carbon supports plays a critical role in both the nanoparticle nucleation (when prepared in‐situ) and anchoring of metal nanoparticles (when prepared ex‐situ). It is well established that defects within graphite‐like structures—particularly those located at the edges of crystallites or as hole‐type imperfections on basal planes—serve as favorable sites for nanoparticle anchoring with ex‐situ synthesis,^[^ ^45^ ^]^ or for nucleation during in‐situ synthesis.^[^ ^58^ ^]^ These defect‐rich spots, often located within microporous voids between interconnected crystallites, create surface regions well‐suited for accommodating Pt nanoparticles.^[^ ^41^ ^]^

Doping carbon with heteroatoms, particularly nitrogen, has been shown to increase the concentration of defects and help promote nucleation or anchoring.^[^ ^45^, ^59^, ^60^ ^]^ Nitrogen doping can be achieved either during synthesis, using nitrogen‐ and carbon‐containing precursors,^[^ ^61^ ^]^ or post‐synthetically by treating carbon materials with common dopants such as ammonia, ammonium nitrate and urea, with pre‐oxidation treatments typically facilitating effective doping.^[^ ^45^ ^]^ The latter approach is particularly useful for isolating the effects of heteroatom incorporation, ideally allowing for controlled modifications in surface chemistry without introducing substantial changes in structural features like crystallinity or porosity.^[^ ^45^ ^]^ Still, the doping process often involves high‐temperature treatments that can unintentionally alter the carbon's morphology and pore structure, complicating direct attribution of performance changes solely to surface chemistry.

During ex‐situ Pt deposition, where nanoparticles are introduced to the support after synthesis, the interaction is largely governed by electrostatic forces modulated through pH adjustment. The introduction of nitrogen functional groups alters the surface charge characteristics of the carbon, thereby influencing deposition behavior. For example, studies have described that with increasing nitrogen content, lower pH values are required to achieve effective nanoparticle anchoring, indicating that nitrogen doping modulates the balance of electrostatic and van der Waals forces.^[^ ^45^ ^]^ This modified interaction is also reflected electrochemically, with Pt ECSA showing a positive correlation with nitrogen concentration, suggesting more uniform nanoparticle coverage due to enhanced support‐nanoparticle affinity.^[^ ^45^ ^]^

With the in‐situ synthesis routes, Pt preferentially nucleates at energetically favorable sites, which can be introduced or enhanced through chemical doping.^[^ ^58^ ^]^ This can improve particle dispersion and minimize agglomeration. However, it also introduces variability in particle size distribution when identical synthesis protocols are applied to supports with differing surface chemistries. Without careful control of synthesis conditions, meaningful comparisons of support effects become difficult. Favorable nucleation effects have been attributed to a range of dopants, including N, P, S, and Fe‐N_4_ moieties.^[^ ^13^, ^62^ ^]^ For example, high‐resolution electron microscopy coupled with electron energy loss spectroscopy (EELS) has revealed that Pt nucleation on N‐doped carbon occurs preferentially near, but not directly on, nitrogen‐rich domains.^[^ ^63^ ^]^ Density functional theory (DFT) studies further reported that Pt binds more strongly to doped regions than to undoped ones, hindering Pt cluster dissolution, migration, and sintering.^[^ ^64^ ^]^

Heteroatom doping has been reported to influence the interaction between the carbon support and the ionomer. There is general agreement about nitrogen‐doped supports, in particular, promoting more uniform ionomer distribution through electrostatic interactions between positively charged nitrogen groups and the ionomer's negatively charged sulfonate side chains.^[^ ^65^, ^66^ ^]^ This enhanced interaction leads to the formation of an ionomer thin film instead of an agglomerate, which reduces mass transport resistance and improves high‐current‐density performance.^[^ ^67^ ^]^ ECSA measurements at varying relative humidities further support this correlation by showing more particles in direct contact with ionomer with the doped support.^[^ ^68^ ^]^ However, the observed effect may also stem from doping‐induced changes in pore structure, which can alter nanoparticle locality.

Disentangling the contributions of heteroatom doping to catalytic activity and durability remains challenging. Three primary mechanisms have been proposed for enhanced oxygen reduction reaction (ORR) activity: i) modulation of the Pt electronic structure through support interactions,^[^ ^69^ ^]^ ii) improved oxygen adsorption due to surface functional groups,^[^ ^70^ ^]^ and iii) increased ECSA resulting in better exposure of Pt sites.^[^ ^59^, ^71^ ^]^ While some studies, including our own, have reported a decrease in specific activity with increasing nitrogen content,^[^ ^45^ ^]^ the literature remains divided, suggesting that the outcome is likely dependent on the properties of the baseline support. Importantly, structural and surface modifications arising from doping can also influence ionomer coverage and Pt nanoparticle locality, further complicating interpretations of activity trends. Regarding durability, enhanced Pt–support interaction due to doping is commonly linked with improved nanoparticle stability.^[^ ^61^, ^72^ ^]^ Some reports also indicate improved resistance to carbon corrosion following nitrogen doping,^[^ ^46^, ^61^ ^]^ although others suggest that dopants may introduce chemically vulnerable sites that accelerate degradation.^[^ ^72^ ^]^

The lack of systematic studies comparing well‐controlled support structures with consistent nanoparticle characteristics underscores the need for further investigation into the isolated effects of heteroatom doping.

## 4 Role of Porosity and Pt Locality

The role of porosity in carbon supports is often discussed in overly simplified terms, typically as a binary classification between low‐ and high‐surface‐area carbons. This distinction is based on the specific surface area of the support calculated from the BET equation and nitrogen adsorption data. Low surface area carbons have a BET surface area below 300 m^2^ g^−1^, whereas so‐called high‐surface‐area carbons can reach values as high as 2000 m^2^ g^−1^.

In a fuel‐cell cathode catalyst, the morphology of the carbon support is important for: i) the Pt interparticle distance that is increased with higher specific surface area, ii) the transport properties of the catalyst layer affected by long‐range porosity and iii) heterogeneity in the local structure affecting both ionomer and water distribution. As a result, examining porosity only through the lens of surface area fails to capture important morphological distinctions. Rather, it is more accurate to consider the pore structures based on their ability to host Pt nanoparticles, and affect gas transport and ionomer accessibility within the catalyst layer. While macro‐ and mesopores may improve gas diffusion, the fabrication process of the electrode itself often exerts a larger influence on long‐range transport than the porosity within the individual support particles. On a more local scale, the size of pore openings can limit ionomer access to certain regions of the support due to steric constraints, altering the electrochemical environment and leading to differences in water retention and condensation at varying relative humidities.

**Figure 4:**
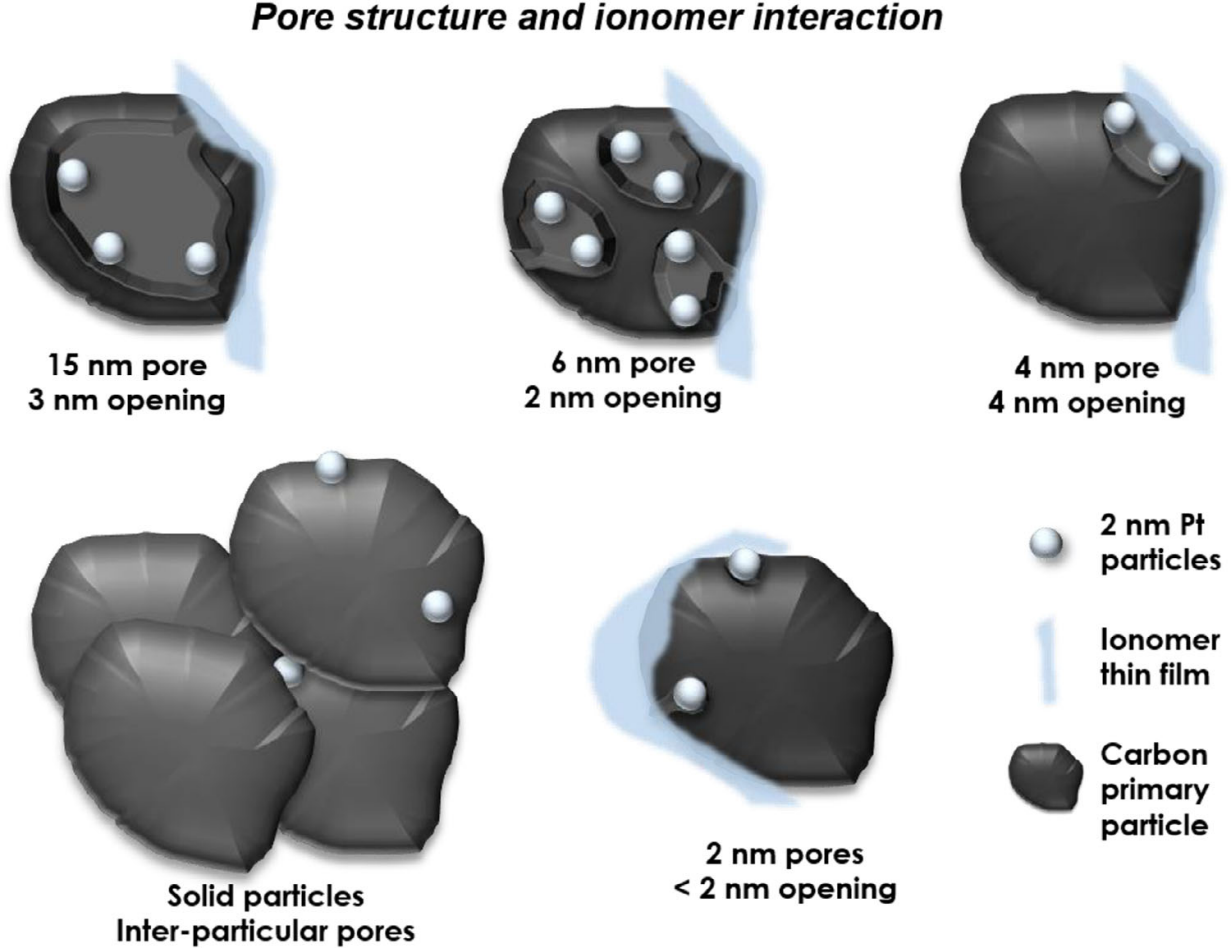
Schematic representation of the different types of porosity and their influence on the accessibility of the ionomer to the nanoparticles.

Given that the BET specific surface area alone cannot fully capture the range of carbon morphologies, a more comprehensive approach to understanding porosity is necessary. In general, porosity is defined as the void volume in a solid material. The International Union of Pure and Applied Chemistry (IUPAC) separates pores by their size as micropores (<2 nm), mesopores (2–50 nm) and macropores (>50 nm).^[^ ^73^ ^]^ However, this sorting of pores by their diameter does not include any information about the pore opening or accessibility of the pore cavity. In the context of a fuel‐cell catalyst support, an “internal pore” can be defined as any void volume that cannot be accessed by the ionomer thin film. Because of its conformational constraints, the ionomer is unable to penetrate pore openings narrower than ≈5 nm. This means that, from the perspective of ionomer–Pt interactions, a large pore (e.g., 20 nm in diameter) with a narrow 3 nm entrance is effectively as inaccessible as a smaller 8 nm pore with a 2 nm opening. In both cases, the pore interior remains out of reach for the ionomer, and Pt nanoparticles located inside such pores cannot be effectively covered or contacted by the ionomer phase. However, these pores will result in very distinct pore size distributions and surface areas. In addition to pores within individual primary particles, the arrangement of these particles into agglomerates and aggregates gives rise to interparticle mesopores. **Figure** **4** illustrates the various types of pores that can exist in carbon supports.

The synthesis pathway used to deposit platinum nanoparticles on the support can determine the locality of the nanoparticles (i.e., their position relative to the pores on a given carbon support). Through wet impregnation of a porous carbon with a Pt salt, followed by thermal reduction, nanoparticles can be preferentially formed inside the pores rather than on the exterior surface of the carbon primary particles. Provided that the Pt precursor salt is highly soluble in the solvent, capillary effects favor its precipitation inside the pores. When Pt‐based nanoparticles are prepared ex situ—for example, colloidally via a polyol‐like method—they preferentially deposit on the most accessible surfaces and are unable to enter pores with narrow openings, regardless of the actual pore size. Many synthesis approaches involve the formation of nanoparticles by wet chemistry with carbon particles present in the reaction medium. In this case, it is the surface chemical environment of the carbon described in Section 2 that governs the nucleation sites, independently of the locality. For many commercial catalysts, the synthesis pathway is unknown, and the spatial distribution of the particles cannot be assumed. Surface‐sensitive STEM or tomography studies are required to determine their locality.

The pore structure of the carbon and the locality of the nanoparticles can drastically change the interaction with the ionomer. To study the effect of this interaction, simply distinguishing between porous carbons and carbons with no porosity in the primary particle (“non‐porous carbons”) is not sufficient. Figure 4 highlights the importance of considering the complexity of the carbon morphology and particle locality when studying ionomer interaction.

### 4.1 Protection from Ionomer

Based on our previous definition of a pore, particles that are localized inside the pores of carbon become inaccessible to the ionomer. The ionomer is a component of the catalyst layer that is very difficult to probe: it is sensitive to X‐rays and electron beams and has very low contrast with the carbon in electron imaging. Neutron‐based techniques provide good contrast with carbon; however, these are complex and far from trivial to implement. The main techniques used to probe the interaction between platinum and ionomer are electrochemical. In PEMFCs, protons travel on the long range via the ionomer network, but can also transport to the active site via water present locally. If nanoparticles are located in pores inaccessible to the ionomer, proton transport can occur only via water. To assess the fraction of platinum that is in direct contact with the ionomer, the ECSA measured in dry conditions is compared to the ECSA measured at high humidity.^[^ ^31^, ^52^, ^74^ ^]^ The difference in surface area between the two reflects the fraction of Pt surface that is only active when water is available to support proton conduction. However, while it is easy to ensure that water is present everywhere by functioning at the saturation limit (100% relative humidity), it is difficult to ensure that no water is present since the fuel cell cannot operate at 0% humidity (dry ionomer does not conduct protons). At low relative humidity (25–30%), the presence of condensed water in the pores by capillary effect can over‐estimate the fraction of particles in direct contact with ionomer. A study by Padgett et. al. combining electron tomography and CO stripping measurements at low relative humidity demonstrates that only at 10% of relative humidity the fraction of probed platinum matches that of the particles outside the pores.^[^ ^31^ ^]^ The porosity and the wettability of the carbon surface strongly influence the condensation of water as a thin film (at lower relative humidity) and by capillarity (at higher relative humidity).^[^ ^75^ ^]^ These phenomena can be understood for each structure through pore modeling and study of water adsorption isotherms, both for the bare carbons and after incorporation of Pt nanoparticles.^[^ ^14^ ^]^ Indeed, the presence of Pt changes the wettability of the surface.^[^ ^75^ ^]^ The modification of the wettability during ageing of the material also needs to be taken into account when trying to assess the amount of particles in direct contact with the ionomer.^[^ ^76^ ^]^

Model studies on thin film and rotating disk electrodes have demonstrated an interaction between the ionomer and the platinum surface that is detrimental to ORR activity.^[^ ^27^, ^28^ ^]^ This translates into an increase of specific activity for the particles that are shielded from direct contact with the ionomer, as demonstrated in membrane electrode assemblies.^[^ ^30^, ^40^, ^77^, ^78^, ^79^ ^]^

The localization of Pt nanoparticles inside the pores adds new complexities regarding transport of protons, oxygen and water. As described previously, in the absence of direct contact with the ionomer, proton conductivity relies on the presence of water near the particles. Then, the performance of the membrane electrode assembly regarding transport will be strongly affected by the relative humidity used. At low relative humidity, a fraction of the particles is inaccessible to protons, which reduces the performance at low current density. At higher relative humidity, however, the pores can fill with liquid water, which enhances the oxygen transport resistance and decreases the performance at high current density.^[^ ^79^, ^80^ ^]^

To obtain the benefits of shielding from the ionomer without limiting the proton and oxygen diffusion to the active sites, so‐called accessible mesopores have been studied.^[^ ^57^, ^78^ ^]^ They present a hierarchical structure with large internal mesopores connected to the external surface via narrow slit micropores formed by cracks between graphitic crystallites.^[^ ^57^ ^]^ Small but numerous openings to a larger pore offer improved transport properties compared to materials with deep mesoporous slits, leading to a more tortuous diffusion pathway. In another approach, Wang et al. used a carbon support with pore structures that allow the deposition of the Pt nanoparticles near the pore entrance to facilitate oxygen transport while protecting from the ionomer.^[^ ^30^ ^]^ Kobayashi et al. demonstrated that in this context, an excess of ionomer can block the pores, decreasing transport properties and performance at intermediate current density.^[^ ^79^ ^]^

In the case of Pt‐alloy nanoparticles, shielding from the ionomer could reduce the local acidity and limit dissolution of the alloying metal. However, it was demonstrated that the dealloying process was occurring similarly for both particles inside and outside of the pores.^[^ ^52^ ^]^ Specifically, this study relied on electron tomography coupled with EELS elemental mapping to assess the aging of the PtCo nanoparticles.^[^ ^52^ ^]^

The placement of Pt nanoparticles inside the pores of the carbon can therefore be highly beneficial for the performance at the beginning of life since it prevents poisoning from the ionomer. However, it can bring transport limitations if the pore entrance is too long and narrow, and/or if the pore is too small, inducing condensation and oxygen transport limitations. Placement of Pt nanoparticles inside the pores can also limit Pt utilization at lower relative humidity.

### 4.2 Protection from Coalescence in Pores

**Figure 5:**
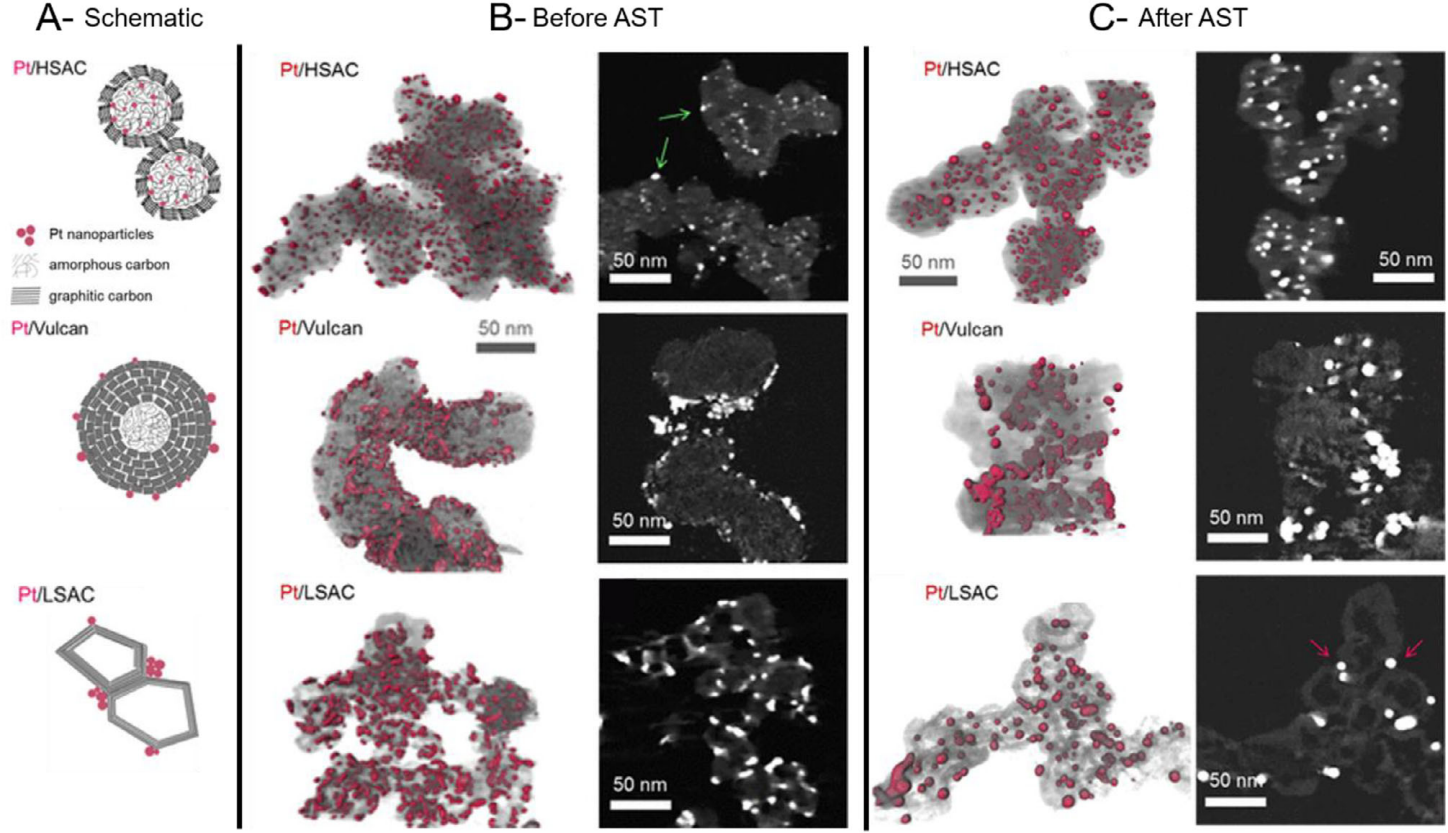
A) Illustrations of the Pt nanoparticle dispersions of commercial materials on Ketjenblack EC300J (HSAC), Vulcan XC72 (Vulcan) and graphitized Ketjenblack (LSAC) with 40 wt% loadings. B) and C) are 3D reconstructions (segmented Pt surfaces appear in red over gray/translucent C volumes) and representative cross‐sectional z‐slices of volumes from electron tomography of as‐prepared Pt/C catalysts (B) and after AST of the MEA. Adapted with permission from Figures 1, 2 and 4 of Ref. [39] © 2017, American Chemical Society.

In addition to the effect on the beginning of life kinetics, the presence of Pt nanoparticles inside of pores has been shown to improve the durability of the material. The particle locality can affect all three degradation mechanisms: coalescence, detachment and Ostwald ripening. Transmission electron tomography is a crucial tool for studying how particles age based on their locality. Padgett et al. demonstrated that, for a porous support with particles inside the pores, Ostwald ripening is the primary mechanism of degradation. However, for nonporous carbons, migration and coalescence dominate.^[^ ^52^ ^]^ The anchoring of the nanoparticles is strongly affected by the chemistry of the carbon surface. Sneed et al. compare the particles' locality for commercial Pt/C materials with Vulcan carbon, Ketjenblack EC300J and graphitized Ketjenblack at different loadings^[^ ^39^ ^]^ using transmission electron tomography (**Figure** **5**). On the graphitized carbon, Pt particles are located at the intersection between graphite planes on the outer surface. On porous and nonporous nongraphitized supports, particles are located outside and inside the pores, respectively. For the two materials with particles outside the pores, particle migration and coalescence are more prevalent than in the material with a porous, nongraphitized structure.^[^ ^39^ ^]^ Using a similar approach, particle coalescence inside the pores of the carbon supports has been demonstrated by Padgett et al., studying the particle size distribution for each family of particles (inside and outside the pores).^[^ ^52^ ^]^ Overall, the pore confinement of the particles seems to restrain the Ostwald ripening and coalescence mechanism to the particles inside the same pore, limiting particle growth and ECSA loss. This pore confinement effect was also assessed through thermal annealing of different catalysts before the electrochemical test. To prepare highly crystalline Pt nanoparticles, Galeano et al. anneal Pt/C materials at 800 °C.^[^ ^81^ ^]^ They observe significant coalescence of the Pt nanoparticles deposited on a nonporous carbon support, while the same degradation mechanism is mitigated when the particles are inside internal mesopores.^[^ ^81^ ^]^

Pore morphology (pore size and opening) and nanoparticle locality have strong effects on the initial performance and durability of the material. To benefit from the porosity, the pores must reduce contact between the nanoparticles and the ionomer, be large enough to optimize oxygen transport and minimize water condensation, and host a limited number of nanoparticles to reduce intrapore coalescence and ripening.

## 5 Perspectives

**Figure 6:**
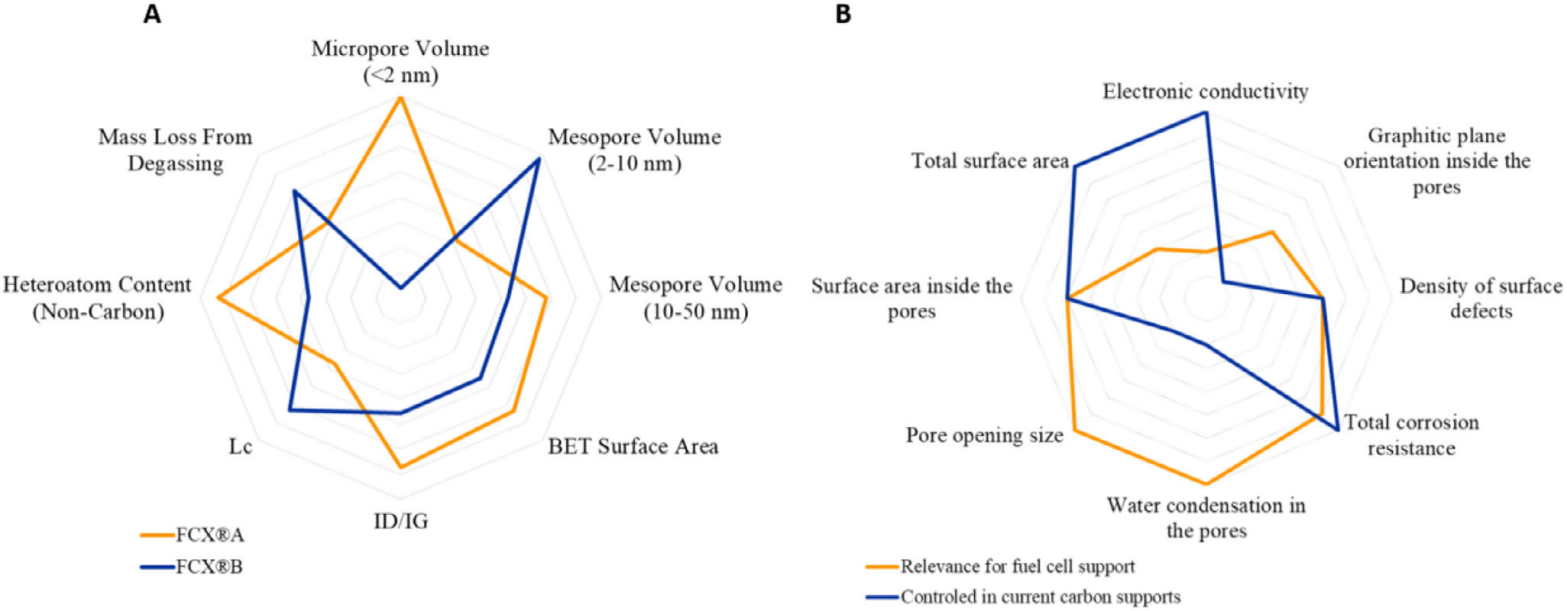
A) Radar plot comparing FCXA and FCXB across various experimentally measured physical and chemical properties. The plot reveals a significant difference in micropore and mesopore (2–10 nm) volume between the two carbons. B) Qualitative visualization of the carbon structure and morphology parameters and how relevant they are for the study of a support for PEMFC cathode, as well as the state of the field regarding the control of this parameter.

The chemical state and morphology of the carbon support have tremendous importance for the performance and durability of the cathode catalyst of PEMFCs. Comparing different carbon materials often makes it difficult to pinpoint which property is responsible for a particular improvement in performance or durability. This review avoids directly comparing different carbon materials and instead highlights the desirable characteristics of carbon supports. A conceptual framework is proposed to understand how these carbon properties influence oxygen reduction in PEMFCs. The presence of graphitic domains improves durability but requires chemical modifications to anchor nanoparticles and mitigate their migration. Porous carbons present the opportunity to shield the nanoparticles from the ionomer. Still, this effect relies on the nanoparticles actually being located inside the pores of the carbon. In such a case, the pore diameter and the size of its openings govern the transport of oxygen and protons, as well as the exposure to the ionomer. The vast diversity of carbon structures makes it essential to carry out tailored characterizations for each type of support in order to truly capture its architecture and understand how it affects the behavior of the catalyst system. **Figure** **6**A shows a visual representation of the parameters commonly used in the literature to study carbon supports. Figure 6B shows other carbon properties that we deem highly important based on this opinion—although not necessarily easily accessible or measurable. We overlap for each parameter the relevance in contributing to an ideal carbon support for fuel cells, and our perception of the degree of control based on current literature. This visualisation highlights how a good control of the precise pore structure of the carbon support, with the arrangement of the graphitic crystals, is important to further push the catalyst materials' performance and durability.

## Conflict of Interest

The authors declare no conflict of interest.
